# *Chlamydia trachomatis *antigens in enteroendocrine cells and macrophages of the small bowel in patients with severe irritable bowel syndrome

**DOI:** 10.1186/1471-230X-10-19

**Published:** 2010-02-16

**Authors:** Aldona Dlugosz, Hans Törnblom, Ghazaleh Mohammadian, Gareth Morgan, Béla Veress, Benjamin Edvinsson, Gunnar Sandström, Greger Lindberg

**Affiliations:** 1 Department of Medicine, Division of Gastroenterology and Hepatology, Karolinska Institutet, Karolinska University Hospital, Huddinge, Stockholm, Sweden; 2 Department of Laboratory Medicine, Division of Pathology, Karolinska Institutet, Karolinska University Hospital, Huddinge, Stockholm, Sweden; 3 Department of Laboratory Medicine, Division of Microbiology, Karolinska Institutet, Karolinska University Hospital, Huddinge, Stockholm, Sweden; 4 Department of Pathology and Cytology, Lund University, University Hospital MAS, Malmö, Sweden; 5 Centre for Microbiological Preparedness, Swedish Institute for Infectious Disease Control, Solna, Sweden

## Abstract

**Background:**

Inflammation and immune activation have repeatedly been suggested as pathogentic factors in irritable bowel syndrome (IBS). The driving force for immune activation in IBS remains unknown. The aim of our study was to find out if the obligate intracellular pathogen *Chlamydia *could be involved in the pathogenesis of IBS.

**Methods:**

We studied 65 patients (61 females) with IBS and 42 (29 females) healthy controls in which IBS had been excluded. Full thickness biopsies from the jejunum and mucosa biopsies from the duodenum and the jejunum were stained with a monoclonal antibody to *Chlamydia *lipopolysaccharide (LPS) and species-specific monoclonal antibodies to *C. trachomatis *and *C. pneumoniae*. We used polyclonal antibodies to chromogranin A, CD68, CD11c, and CD117 to identify enteroendocrine cells, macrophages, dendritic, and mast cells, respectively.

**Results:**

*Chlamydia *LPS was present in 89% of patients with IBS, but in only 14% of healthy controls (p < 0.001) and 79% of LPS-positive biopsies were also positive for *C. trachomatis *major outer membrane protein (MOMP). Staining for *C. pneumoniae *was negative in both patients and controls. *Chlamydia *LPS was detected in enteroendocrine cells of the mucosa in 90% of positive biopsies and in subepithelial macrophages in 69% of biopsies. Biopsies taken at different time points in 19 patients revealed persistence of *Chlamydia *LPS up to 11 years. The odds ratio for the association of *Chlamydia *LPS with presence of IBS (43.1; 95% CI: 13.2-140.7) is much higher than any previously described pathogenetic marker in IBS.

**Conclusions:**

We found *C. trachomatis *antigens in enteroendocrine cells and macrophages in the small bowel mucosa of patients with IBS. Further studies are required to clarify if the presence of such antigens has a role in the pathogenesis of IBS.

## Background

The irritable bowel syndrome (IBS) is a common disorder that may affect as many as 9%-15% of the population in Western countries [1-3]. IBS is characterized by abdominal pain and disturbed bowel function in the absence of a detectable organic disease, which may explain the symptoms [4]. The presence of disturbed gut function in IBS may indicate an underlying pathology in control systems or effectors of the gut. We have previously reported lymphocytic infiltration and neuron damage in myenteric ganglia when full-thickness biopsies from the jejunum in 10 patients with severe IBS were investigated [5], and there are many reports that have highlighted signs of an activated immune system as a putative pathogenetic mechanism in IBS (for review see de Giorgio [6]). However, the aetiology of observed immune activation remains unsolved.

Studies have repeatedly indicated that IBS can arise after an acute gastroenteritis. The underlying mechanism of post-infectious IBS has not been established but ongoing inflammation appears to play a role, with an increase in enteroendocrine cells, lymphocytes, mast cells, and proinflammatory cytokines (for review, see Spiller[7]). However, in a recent study of ours the actual agent causing gastroenteritis was not a predictor of risk for IBS [8]. Consequently, a host factor, such as a pre-existing chronic infection with a different microbe than the agent causing gastroenteritis, might explain the development of IBS. We presumed that a candidate agent should be compatible with an asymptomatic carrier-ship, have a preference for female gender, and have the ability to become persistent and to live in bowel epithelium. There are some observations to support the idea that a persistent infection with *Chlamydia trachomatis *might constitute such a host factor. Trachoma-related blindness is 2-4 times more likely to affect females compared to males [9]. It is known that IBS occurs in 35%-50% of females with chronic pelvic pain syndrome, which is believed to often be caused by chronic infection with *C. trachomatis *[10-12]. A previous attempt to link *C. trachomatis *to IBS using serum IgG antibodies failed [13], but IgG antibody patterns may be insufficient to rule out persistence of *Chlamydia *due a dominating cellular immune response to infection [14,15]. Since we had previously found inflammation in mucosa and enteric ganglia of the jejunum in patients with IBS we decided to reanalyze archived biopsy material to find out if Chlamydia antigens are present in the small bowel in this group of patients.

## Methods

### Patients

All patients fulfilled Rome-II criteria for IBS [4]. A total of 65 patients (61 females and 4 males) with a median age of 48 (range 22-67) years and a median duration of IBS symptoms of 6.5 years (range 0.6-33.2 years) were investigated. Diarrhoea predominant IBS was present in 21 patients (32%), 22 patients (34%) had constipation predominant IBS and 22 patients (34%) had IBS with alternating bowel habits. All patients had severe symptoms of IBS [5] and 26 patients also exhibited abnormalities on small bowel manometry, thus qualifying for a pathophysiological diagnosis of enteric dysmotility [16]. Full thickness jejunum biopsies had been taken in 60 of our patients. Previous histopathological analysis had revealed neuropathic changes in 58 patients [5,17,18]. Neuropathy was associated with low-grade inflammation (LG = lymphocytic ganglionitis) in 46 patients and 20 of these also exhibited increased numbers of intraepithelial lymphocytes (LEG = lymphocytic epithelio-ganglionitis), whereas 12 patients had degenerative neuropathy (DN) without inflammation. Deficient staining for alpha-actin without neuronal damage was observed in 2 patients.

### Controls

The control group comprised 42 persons (29 females) in whom IBS and all other functional bowel disorders had been excluded by medical interview and a validated questionnaire for the Rome-II symptom criteria. Ten controls (7 females) were obese but otherwise healthy (BMI mean = 42.8, SD = 4.3). The rest of the control group (32 persons, 22 females) consisted of healthy volunteers. The median age of the controls was 36 (range 19-60) years.

### Full thickness jejunum biopsy

Previously obtained full-thickness biopsy specimens were available in 60/65 patients. The biopsies had been taken from the proximal jejunum using a laparoscopy-assisted procedure described by Tornblom et al[5] Ten obese controls underwent full thickness biopsy of the jejunum at the time of gastric by-pass surgery.

### Mucosa biopsy

Mucosa specimens from the proximal jejunum were taken with a Watson capsule in 32 controls and 6 patients. The capsule was swallowed by the subject and brought by peristalsis to a position distal to the ligament of Treitz as determined by fluoroscopy. We analyzed archived endoscopic mucosa biopsies from the duodenum of 20 patients and in 15 of these full thickness biopsies were also available. In 2 patients we analyzed mucosa biopsies from both the jejunum and the duodenum.

### Immunofluorescence

Immunofluorescence was performed using a genus-specific mouse monoclonal antibody to *Chlamydia *lipopolysaccharide (LPS)-FITC conjugated with Evans blue (RDI-PROAC1FT, Fitzgerald Industries International, Concord, MA, USA) and *C. trachomatis *major outer membrane protein (MOMP) as primary antibody (GeneTex, San Antonio, TX, USA) with a polyclonal rabbit anti-mouse antibody-FITC conjugated (Dako, Glostrup, Danmark) as the secondary antibody. We used cultured HeLa-cells infected with *C. trachomatis *as positive control and uninfected cells as negative control. We used a species-specific mouse monoclonal antibody for *C. pneumoniae *as primary antibody (GeneTex, San Antonio, TX, USA) with a polyclonal rabbit anti-mouse antibody-FITC conjugated (Dako, Glostrup, Denmark) as the secondary antibody. The presence of *Chlamydia *LPS was also evaluated using immunohistochemistry with a polyclonal rabbit antibody to *C. trachomatis *LPS (Fitzgerald Industries International, Concord, MA) and an immunoenzymatic assay with Streptavidin-biotin complex (Dako, Glostrup, Danmark).

Enteroendocrine cells were identified using rabbit polyclonal antibodies to Chromogranin A (Abcam, Cambridge, UK), and we used rabbit polyclonal antibodies to CD117 (Dako, Glostrup, Denmark) for mast cells, rabbit polyclonal antibodies to CD68 (Santa Cruz Biotechnology, Santa Cruz, CA, USA) for macrophages and rabbit monoclonal antibodies to CD11c (Abcam, Cambridge, UK) for dendritic cells. Goat anti-rabbit antibodies conjugated with Alexa Fluor 568 or Alexia Fluor 350 (Invitrogen, Carlsbad, CA, USA) were used as secondary antibody for all of the rabbit antibodies.

Stained sections were examined using a Universal Laser Scanning Confocal Microscope System Leica TCS and a Fluorescent Microscope System Leica DMRXA (Leica Microsystems, Wetzlar, Germany). Two independent investigators (AD and GM), who were unaware of clinical data, made the final assessment of slides. The slides were reviewed by a third investigator (BV). The immunofluorescence stainings were considered positive if more than one cell showed fluorescence. If only one positive cell was found, the staining-procedure was repeated. The case was regarded positive if the colour signal was again present, otherwise the case was recorded as negative. Double immunofluorescence stainings were also performed for the identification of LPS-positive cells (LPS with chromogranin, CD68, CD117, or CD11c, respectively).

### Western blotting

Snap-frozen biopsies from 4 patients that were positive for *Chlamydia *LPS staining, were examined by Western blotting. HeLa cells infected with *C. trachomatis *served as positive control and we used non-infected HeLa cells as negative control. Equivalent amounts of protein from each specimen were loaded onto a sodium dodecyl sulphate-polyacrylamide gel. After electrophoresis, samples were transferred to nitrocellulose membranes. We used a mouse monoclonal antibody to *C. trachomatis *LPS (AbD Serotec, Oxford, UK) as primary antibody and goat anti-mouse antibody conjugated to horseradish peroxidase (BioRad, Herculaes, CA, USA) as secondary antibody. For MOMP we used the same primary antibody as for immunofluorescence.

### MicroLaser system

We used Laser Microdissection Pressure Catapulting (LMPC) for laser based non-contact extraction of tissue areas in paraffin embedded biopsies from 6 patients. Regions of interest were manually delineated using fluorescence microscopy and the LMPC software. Tissue collection was achieved by laser cutting along the delineation lines to separate tissue of interest from surrounding regions, and secondly the laser catapulted the tissue of interest up into the lid of an Eppendorf cap containing sterile water. DNA was extracted using the QIAamp DNA mini kit, according to the instructions of the manufacturer, and analyzed by PCR.

### Real-time PCR

In the present study, we used the real-time PCR assay developed by Everett et al. [19], which amplifies 23S ribosomal DNA, and detects all members of the family *Chlamydiaceae*. An internal amplification control was included to monitor possible inhibition of the PCR. DNA from frozen biopsies, taken from 4 patients previously positive for *Chlamydia *LPS staining, was extracted using a Qiagen minikit according to the tissue protocol (Qiagen, Solna, Sweden). Extracted DNA was quantified and quality controlled using a Nanodrop spectrophotometer (Nanodrop Technologies, Wilmington, DE, USA) before being subjected to PCR.

### Transmission electron microscopy

Biopsies from the distal duodenum of 4 patients that were positive for *Chlamydia *LPS staining were fixed according to a procedure described before [20]. Semi-thin sections were cut and stained with toluidin blue and used for light microscopic analysis. Ultra-thin sections were contrasted with uranyl acetate followed by lead citrate and examined in a Tecnai 10 transmission electron microscope at 80 kV. Digital images were taken by using a MegaView III digital camera (Soft Imaging System, Münster, Germany).

### Statistical analysis

We used logistic regression with age and gender as covariates for calculation of odds ratios and p-values for comparisons of proportions. The size of the study group was determined from the assumption that LPS positivity would be no greater than 20% among controls. In order to detect a risk factor with an odds ratio of at least 6 at p < 0.01 with power >90% we needed to include at least 37 patients and 37 controls (two-sided test).

### Ethical considerations

All parts of the study were approved by the Regional Ethical Review Board in Stockholm. Informed consent was obtained from all patients and controls at the time of biopsy taking.

## Results

**Figure 1:**
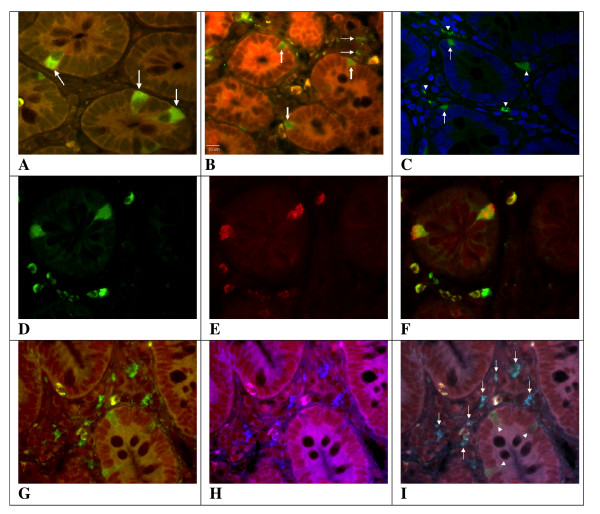
**Fluorescent microscope images of small bowel preparations from patients with IBS A**. *Chlamydia *LPS in EEC-like cells with apical nuclei and strong basal immunofluorescence (arrows). (Monoclonal FITC-conjugated antibody with Evans blue; original magnification × 63). **B**. *Chlamydia *LPS in a few cells within the epithelium (thick arrows) and *l. propria *(thin arrows). (Monoclonal FITC-conjugated antibody with Evans blue; original magnification × 63). **C**. *Chlamydia trachomatis *MOMP-positive immunofluorescence within 2 EEC-like cells (arrows) and 4 cells within *l. propria *(arrowheads). (Mouse MOMP-antibody and FITC-conjugated rabbit anti-mouse antibody; original magnification × 63). Hoechst (DAPI conjugated) for nuclear staining. **D-F**. Immunostainings for **(D) ***Chlamydia *LPS (FITC, green), (**E**) chromogranin A (Alexia 568, red), and (**F**) merged showing co-localisation of chromogranin A and *Chlamydia *LPS in enteroendocrine cells and *Chlamydia *LPS in *l. propria*. **G-I**. Immunostainings for (**G**) *Chlamydia *LPS (FITC, green,) (**H**) CD68 (Alexia 350. blue), and (**I**) merged showing co-localisation of CD68 and *Chlamydia *LPS in macrophages (arrows). Three enteroendocrine cells are also positive for *Chlamydia LPS *(arrowheads).

Immunofluorescence (IF) staining showed that 53/60 patients were positive for *Chlamydia *LPS in full thickness jejunum biopsies. Positive staining occurred in a few cells both within the epithelium and in *lamina propria *(Figure 1). No *Chlamydia *LPS-positive cells were found in the submucosa and *muscularis propria *or the enteric nervous system. Since we had full thickness biopsies from only 10 controls, we investigated if the finding of LPS-positive cells could be reproduced in biopsies from small bowel mucosa. We investigated mucosal biopsies from 24 of our patients with IBS and 32 controls. Staining of slides from these biopsies yielded similar rates of positive stainings: 21/24 (88%) patients were positive for *Chlamydia *LPS again in a few cells within the epithelium and in *l. propria*. In 19 patients biopsies had been taken with a time interval of more than 1 year. *Chlamydia *LPS was present in biopsies with a median time difference of 5.2 (range 1-11) years. Overall, 58/65 (89%) patients were classified as *Chlamydia *LPS positive. Six women and one man with IBS were negative in all biopsies.

In 90% of positive biopsies from patients *Chlamydia *LPS was localised to mucosal cells at the level of the crypts. Double staining with antibodies to chromogranin-A showed that *Chlamydia *LPS was present in enteroendocrine cells within the epithelium (Figure 1). *Chlamydia *LPS was found in *l. propria *cells in 69% of the biopsies from patients. Double staining with antibodies to CD117, CD11c and CD68 revealed that in this location *Chlamydia *LPS was present in macrophages. Seventy-nine percent of *Chlamydia *LPS positive biopsies were also positive to *C. trachomatis *MOMP (Figure 1). Staining for *C. pneumoniae *was negative in all patients.

Only 6/42 (14%) controls (5 women) were positive for *Chlamydia *LPS (6 in *l. propria *macrophages and 2 in enteroendocrine cells) and 2/6 LPS-positive controls were positive to *C. trachomatis *MOMP. No biopsies from controls were positive for *C. pneumoniae*. Slides from 10 patients who were positive for *Chlamydia *LPS in immunofluorescence and 10 negative controls were studied using immunohistochemistry with polyclonal antibodies to *Chlamydia *LPS and streptavidin-biotin complex. *Chlamydia *LPS was found in enteroendocrine cells and/or macrophages in all cases whereas all controls remained negative.

The prevalence of *Chlamydia *LPS was much higher in patients with IBS (58/65) than it was among controls (6/42). The odds ratio, corrected for differences in age and gender distributions, for mucosal *Chlamydia *LPS being indicative for presence of IBS was 43.1 (95% CI: 13.2-140.7). Biopsies were analysed by two independent investigators unaware of clinical data. The agreement between the two investigators regarding individual biopsies was 94%, whereas their agreement regarding patient classification was 100%.

We found no correlation between presence of *Chlamydia *antigens and the type of neuropathy in patients with full thickness biopsies. Although the majority of patients had neuropathy with inflammation, *Chlamydia *antigens were common also among those who had neuropathic changes without inflammation (LEG: 19/20 = 95%; LG 23/26 = 88%; DN 9/12 = 75%). We found no difference between IBS subgroups with regard to positivity for Chlamydia antigens and 19/21 with D-IBS, 22/22 with C-IBS, and 17/22 with A-IBS were LPS positive.

**Figure 2:**
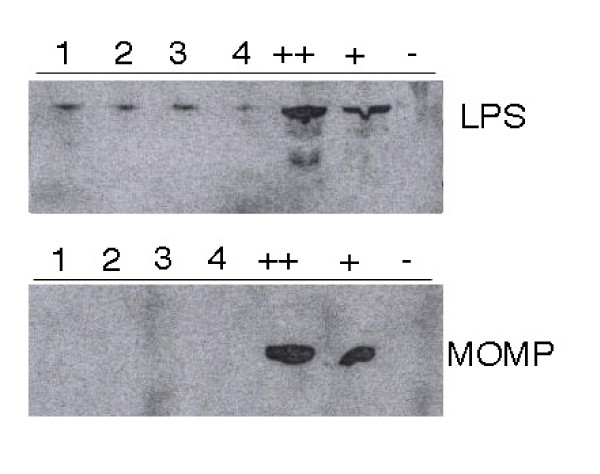
**Western blot**. Western blot analysis of small bowel biopsies from 4 IBS patients against mouse immune sera to LPS and MOMP, respectively. 1-4 = IBS patients, ++ = HeLa cells infected with *C. trachomatis *(10 μl), + = HeLa cells infected with *C. trachomatis *(5 μl), - = non-infected HeLa cells.

**Figure 3:**
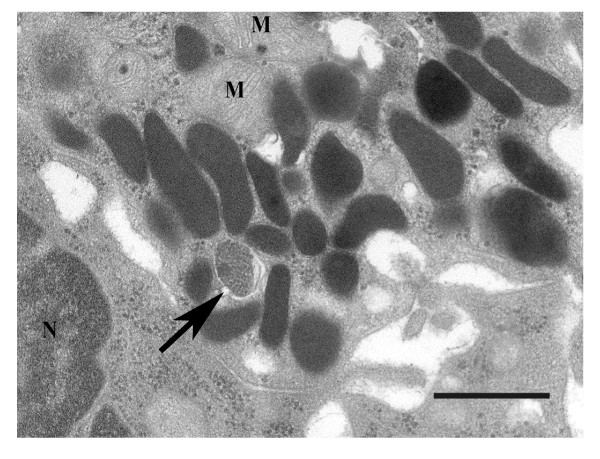
**Transmission electron microscopy**. Electron micrograph of the cytoplasm of an enteroendocrine cell from a patient with IBS showing a structure resembling an intermediate body of Chlamydia (arrow) with characteristic condensed nucleoids. Dark homogenous structures are granules. M = Mitochondria, N = Nucleus, Bar = 0.5 μm.

New biopsies were taken from the duodenum mucosa in 4 LPS-positive patients (Table 1). We confirmed the presence of *Chlamydia *LPS antigen in patients with Western blotting but the new biopsies were negative for *C. trachomatis *MOMP both in immunofluorescense and Western blot (Figure 2). Electron microscopy revealed small oval structures resembling intermediate bodies of *Chlamydia *with characteristic condensed nucleoids of nucleic acid (Figure 3). Mitochondria were observed surrounding the inclusions but did not appear to be in close association with the inclusions. We were unable to confirm the presence of *Chlamydia *DNA in the same biopsies using 23S ribosomal DNA as target. Results were negative for *Chlamydia *DNA also when we analyzed micro-dissected *Chlamydia *LPS positive cells.

**Table 1:** Onset of disease and results from immunofluorescence staining for *Chlamydia *LPS and *C. trachomatis *MOMP, Western blot, polymerase chain reaction (PCR), and electron microscopy (EM) in 4 patients selected for new biopsies

Patient	Onset of IBS symptoms	Archive biopsies			New biopsies 2007		Western blot 2007		PCR	EM
				
		Year	LPS	MOMP	LPS	MOMP	LPS	MOMP	2007	2007
1	1994	1996	+	+	+	-	+	-	-	+

2	1985	2000	+	+	+	-	+	-	-	+

3	1968	2001	+	+	+	-	+	-	-	+

4	1977	1997	+	+	+	-	+	-	-	+

## Discussion

We report the novel finding of chlamydial antigens in enteroendocrine cells and macrophages of the small bowel mucosa in patients with severe IBS. LPS and MOMP antigens were detected in mucosa and *l. propria *of the small bowel in a large majority of patients with IBS, but rarely so in healthy controls. The odds ratio for mucosal *Chlamydia *LPS being indicative for presence of IBS is much higher than any previously described pathogenetic marker in IBS [21]. These findings raise several questions. The first is whether or not observed immunofluorescence findings represent presence of bacterial antigens. The specificity of the LPS antibody is important in this respect. There is a risk that antibodies may give rise to unspecific binding but such binding should not differ between patients and controls. We therefore think that unspecific binding is an unlikely explanation for our findings. It has previously been shown that this monoclonal anti-LPS antibody does not bind to environmental *Chlamydiae *[22,23]. The genus-specific LPS epitope is not shared by other known gram-negative bacteria and monoclonal antibodies do not bind LPS of those organisms [24]. Thus, positive staining of LPS can be considered as a marker of past or present *Chlamydia *infection. We used different techniques to visualize Chlamydia LPS. In addition to immunofluorescence with a FITC-conjugated monoclonal antibody, we used a polyclonal antibody to Chlamydia LPS and the standard streptavidin-biotin technique for light microscopy. The latter technique also showed presence of antigen in the same cell types. We used Western blot on new biopsies from a limited number of patients and confirmed that Chlamydia LPS was present in the tissue samples from these patients. These findings further support the hypothesis that observed antigens have a bacterial origin.

The second question concerns the identity of the species involved. We used a genus-specific antibody to *Chlamydia *LPS and species-specific antibodies to *C. pneumoniae *and *C. trachomatis *MOMP. Since we found positive staining for *C. trachomatis *MOMP in 79% of *Chlamydia *LPS biopsies and none of them was positive for *C. pneumoniae *we hypothesized that the origin of these antigens is a past or present infection with *C. trachomatis*.

The third question is if observed antigens represent an ongoing infection or not. We investigated archived biopsy material from the small bowel and this puts certain limits on our ability to ascertain the underlying cause for observed findings as well as their specificity. To confirm and strengthen our immunohistochemical findings we used antibodies to different antigens and also different methods of visualisation. In a limited number of cases we had the opportunity to take new biopsies for more advanced analyses using molecular biology techniques, proteomics and electron microscopy. Whereas electron microscopy showed presence of *Chlamydia*-like organisms in the cytoplasm of enteroendocrine cells, similar to those described in persistent *C. trachomatis *infection [25], we were unable to detect chlamydial DNA in a small number of patients using standard extraction and amplification protocols. It is possible that the standard methods we used for nucleic acid retrieval were inadequate for the detection of a persistent infection [26]. Another explanation for the contradictory results could be that chlamydial antigens were remainders of a past, but no longer present infection [27]. The latter seems unlikely in light of the long-term presence of *C. trachomatis *antigens observed in several of our patients. Long-term presence of antigens is more likely to be attributable to replicating *Chlamydiae *residing in the diseased tissue [28]. At present, however, we cannot determine if patients with severe IBS have an ongoing intestinal infection with *C. trachomatis *or not. Neither can we explain the presence of inflammation in myenteric ganglia or neuropathy by the finding of *Chlamydia *antigens in enteroendocrine cells. A possible link between the presence of *Chlamydia *antigens in enteroendocrine cells and myenteric inflammation could be autoimmune mechanisms related to heat shock protein 60 [29], but at this time such a mechanism remains purely speculative.

We found *Chlamydia *antigen also in macrophages. It is known that *Chlamydiae *become spontaneously persistent following the infection of monocytes and the monocyte is the common host cell for *Chlamydiae *during persistent infection [30-32]. *Chlamydiae *may participate in the maintenance of local immunological response and inflammation via infected monocytes/macrophages and also use them to spread infection to other organs [33].

Some of our *Chlamydia *LPS positive patients did not show the presence of MOMP antigen. *Chlamydiae *down-regulate the major outer membrane protein (MOMP) expression in the persistent state [34]. Attenuated synthesis of MOMP, usually considered the immuno-dominant surface epitope of the organism, in combination with its almost exclusively intracellular location during persistence may help to provide some relative invisibility from immune surveillance [34].

The presence of *C. trachomatis *antigens in small bowel mucosa has not been demonstrated in IBS patients before. However, it is known that *Chlamydiae *can persist in the intestinal mucosa of certain animals for a long time without infection-induced inflammation [35]. The specificity of *C. trachomatis *antigens for IBS compared to other gastrointestinal diseases is unknown. We did not have access to biopsies from the upper small intestine of patients with for example Crohn's disease or ulcerative colitis. At the same time, it is difficult to rule out the co-existence of IBS with such diseases and this might blur the results of such a comparison. We therefore decided to compare our patients with severe IBS to healthy controls in which IBS and other motility disorders could be excluded.

The finding of *C. trachomatis *antigens in enteroendocrine cells makes it tempting to suggest a novel pathogenetic mechanism in IBS. Enteroendocrine cells play a pivotal role in the control of gut motility and secretion and increased numbers of enteroendocrine cells have been detected in patients that developed IBS after an acute gastroenteritis [36]. It is unknown if infection alters the function of enteroendocrine cells in man but animal experiments using different models of enteric infection have shown pronounced changes in both numbers of enteroendocrine cells and their function [37,38]. Serotonin-producing enteroendocrine cells may present an ideal location for *Chlamydia *due to their abundance of tryptophan. Tryptophan is required for normal development in *Chlamydia *species and tryptophan metabolism has been implicated in *Chlamydia *persistence and tissue tropism [39]. We hypothesize that *Chlamydia *actively may enter EEC searching for tryptofan. Infection may impair EEC serotonin and hormone secretion and cause motility disturbances but further studies on enteroendocrine cell lines or cultured EEC are required for elucidating such a mechanism.

## Conclusions

We found chlamydial antigens in enteroendocrine cells and macrophages of the small bowel mucosa in 89% of patients with IBS but in only 14% of controls. Even though we were unable to prove the presence of viable *Chlamydia*, lack of positive PCR being the major obstacle, the evidence for an intracellular organism or at least a protein structure with an antigen in common with *Chlamydia *in patients with IBS is strong. Our results suggest that the presence of *C. trachomatis *antigens in enteroendocrine cells may be involved in the pathogenesis in IBS.

## Abbreviations

CI: confidence interval; DN: degenerative neuropathy; DNA: deoxyribonucleic acid; EEC: enteroendocrine cells; FITC: fluorescein (-isothiocyanate); IBS: irritable bowel syndrome; LEG: lymphocytic epithelio-ganglionitis; LG: lymphocytic ganglionitis; LPS: lipopolysaccharide; LMPC: laser microdissection pressure catapulting; MOMP: major outer membrane protein: PCR: polymerase chain reaction.

## Competing interests

The authors declare that they have no competing interests.

## Authors' contributions

AD participated in the design and coordination of the study, carried out the immunohistochemical analyses, performed literature review, and drafted the manuscript. HT participated in collecting clinical data and biopsies from patients. GhM obtained clinical data and biopsies from healthy controls. GM participated in the analysis of immunohistochemical stainings. BV performed the histopathological analysis of full thickness bowel biopsies and reviewed immunohistochemical stainings. BE carried out nucleic acid amplification analyses and Western blot. GS participated in the design of the study and the analysis of data. GL collected clinical data and biopsies from patients, performed the statistical analysis, participated in the design of the study and supervised the study. All authors read and approved the final manuscript.

## Pre-publication history

The pre-publication history for this paper can be accessed here:

http://www.biomedcentral.com/1471-230X/10/19/prepub
